# Inhibition of Proinflammatory Cytokines in *Cutibacterium acnes*-Induced Inflammation in HaCaT Cells by Using *Buddleja davidii* Aqueous Extract

**DOI:** 10.1155/2020/8063289

**Published:** 2020-04-21

**Authors:** Anh Thu Nguyen, Ki-young Kim

**Affiliations:** Graduate School of Biotechnology, Kyung Hee University, Giheung, Yongin-si, Gyeonggi-do 1732, Republic of Korea

## Abstract

Acne is an inflammatory skin disorder; although some anti-inflammatory medicines for treating acne are available in a market, they have considerable side effects; therefore, new treatment options are needed. In the present study, among the 16 aqueous extracts of plants collected from Jeju Island in Korea which are used to test anti-inflammatory activity, *B. davidii* showed the strong decline of the proinflammatory cytokine expression against the inflammatory process caused by *C. acnes* in Human HaCaT keratinocyte cells. *B. davidii* downregulated the expression of 57% of COX-2, 41% of iNOS, and proinflammatory cytokines 29% of TNF-*α*, 32% of IL-1*β*, 21% of IL-6, and 35% of IL-8. Furthermore, *B. davidii* inhibited NF-*κ*B and MAPK signaling cascades in keratinocytes that activated by toll-like receptor 2 (TLR-2) in response to *C. acnes*. Given those results, *B. davidii* is a potential agent to reduce the proinflammatory cytokine expression against *C. acnes*-induced inflammation and might provide an alternative to the current medications.

## 1. Introduction

Acne is a chronic inflammatory disorder of the sebaceous follicles on the chin, cheek, forehead, and back. Cysts and scarring can occur when the disease is severe. Although acne is a skin disease, it can lead to psychological issues that affect patient quality of life. In acne pathogenesis, *C. acnes* has been identified as one of the main causative agents. *C. acnes*, a commensal Gram-positive anaerobic, colonizes the duct of the sebaceous follicle, causing an innate immune response [1].

Inflammation is the body's initial complex response to infection. The processes of inflammation caused by *C. acnes* begin when peptidoglycan (PGN) and lipoteichoic acid (LTA) recognized by binding to toll-like receptors (TLRs) activate the TLR signaling pathway and trigger important intracellular signaling pathways including the mitogen-activated protein kinase (MAPK) and nuclear factor kappa-B (NF-*κ*B) pathways. This causes nuclear translocation of transcription factors, such as activator protein-1 (AP-1) and NF-*κ*B, ultimately leading to production of a variety of proinflammatory cytokines including tumor necrosis factor (TNF) *α*, interleukin (IL) 1*β*, IL-8, and IL-6 [2, 3].

Adverse effects associated with anti-inflammatory drugs have become increasingly common and include increased risk of gastrointestinal ulcers, bleeding, heart attack, and kidney disease associated with nonsteroidal anti-inflammatory drugs (NSAIDs) [4]. Some anti-inflammatory drugs interfere with deactivated and hyperactivated cellular signaling networks or transcription factors [4]. Therefore, to limit adverse effects, the next generation of anti-inflammatory drugs needs to target either enzymes or signaling pathways that are only operational in affected tissues or a small subset of inflammatory target genes.

Medicinal plants have long been used as sources of therapeutically active compounds, which is also a popular traditional medicine to treat inflammatory diseases. The use of traditional medicines for treatment of inflammation could provide safer alternatives compared with existing conventional treatments. Evidence suggests that herbal medicines have a lower rate of side effects compared with conventional medicines [5]. Furthermore, medicinal plants used for dermatological purposes with low or no side effects are gaining popularity in both traditional medicine and the cosmetic industry. *Buddleja davidii* is a perennial herbaceous plant that belongs to the Scrophulariaceae family with a pan tropical distribution across South Asia, Africa, and America. Its roots, leaves, and flowers are used in folk medicine for antioxidant, anti-inflammation, antiviral, antiplasmodium, and antifungal effects [6, 7].

The purpose of the present study was to determine the anti-inflammatory activity of *B. davidii* against *C. acnes* infection and to elucidate its mechanism of action in human keratinocyte cell line.

## 2. Materials and Methods

### 2.1. Preparation of Bacteria

*C. acnes* (CCARM9009) were purchased from the Culture Collection of Antimicrobial Resistant Microbes (CCARM) at Seoul Women's University in Korea. *C. acnes* were grown in Reinforced Clostridial Medium (RCM) agar (Becton, Dickinson and Company, USA) for 48–72 hours at 37°C under anaerobic conditions in a GasPak™ EZ anaerobic container system (Becton, Dickinson and Company, USA). *C. acnes* were cultured in RCM broth to 100 MOI and then heat-killed at 80°C for 30 minutes before use in further experiments [2].

### 2.2. Plant Material and Extraction

Candidate plants, the herb medicine with anti-inflammation properties, were obtained from Jeju Island in South Korea. The plants were cleaned with distilled water and then dried using a freeze dryer (iLShin, Korea). After drying, 300 g of the dried herb was extracted three times in 3 liter of distilled water with refluxing at 80°C for 8 hours. 500 ml extract was concentrated under reduced pressure at 50°C using a rotary evaporator (EYELA, Japan) connected to a refrigerated bath circulator (Jeio Tech, Korea) [8]. The decoction was filtered using muslin cloth followed by Whatman (GE Healthcare, USA) grade 1 filter paper, lyophilized using a freeze dryer (iLShin, Korea), and stored at 4°C before further use [9]. To prepare the sample for experiments, the stock of extract powder (10 mg) was dissolved in 1 ml of dimethyl sulfoxide (DMSO).

### 2.3. Cell Culture

Human HaCaT keratinocyte cells [10] were grown in Dulbecco's Modified Eagle's Medium (DMEM) supplemented with 10% fetal bovine serum and 100 U/ml penicillin at 37°C in a humidified incubator under 5% CO_2_.

### 2.4. Quantitative Real-Time PCR

HaCaT cells (seeded at a density of 5 × 10^5^ per well in a six-well plate) were incubated for 24 hours, pretreated with various concentrations (12.5, 25, 50 and 100 *μ*g/ml) of aqueous extract of plants for 1 hour, and subsequently treated with heat-killed *C. acnes* (100 MOI, 5 × 10^7^ CFU per well) for 24 hours. Total RNA was isolated from the HaCaT cells using TRIzol reagent (Life Technology, Thermo Fisher Scientific, USA) according to the manufacturer's protocol. A reverse transcriptase (NanoHelix, Korea) reaction was prepared using 1 *μ*g of RNA to obtain cDNA. The cDNA was used as the template for real-time PCR (qRT-PCR), which was carried out with *QGreen* 2X SybrGreen qPCR Master Mix (CellSafe, Korea). The primer sequences used to detect TLR-2, iNOS, COX-2, TNF-*α*, IL-8, IL-6, IL-1*β*, and GAPDH are listed in Table 1. GAPDH was used as an endogenous control. The delta-delta Cq formula is used to calculate the gene expression.

### 2.5. Western Blot Analysis

HaCaT cells were treated with *B. davidii* aqueous extract and heat-killed *C. acnes* as described above. The cells were then lysed using radioimmunoprecipitation assay buffer (RIPA) lysis buffer containing 150 mM sodium chloride, 1% Triton X-100, 0.5% sodium deoxycholate, 0.1% sodium dodecyl sulfate (SDS), 50 mM Tris (pH 8.0), and complete protease inhibitor cocktail (BIOMAX, Korea). Protein concentration was determined with the Bradford ((Sigma-Aldrich, USA) assay using bovine serum albumin as a standard and detected using a UVITEC imaging system (Uvitec Ltd, UK). Protein samples (50 *μ*g) were separated by sodium dodecyl sulfate-polyacrylamide gel electrophoresis (SDS-PAGE) and transferred to polyvinylidene difluoride (PVDF) membrane. The membranes were blocked with 5% skim milk in TBST buffer (20 mM Tris-HCl, 150 mM NaCl, and 0.1% Tween 20, pH 7.6) for 1 hour at room temperature and then subsequently incubated with primary antibodies including p-NF-*κ*B/NF-*κ*B (sc-101752/8242S), p-p38/p38 (sc-17852/sc-535), p-JNK/JNK (sc-6254/sc-7345), and GAPDH (sc-25778) (Santa Cruz Biotechnology, Inc, USA and Cell Signaling Technology, Inc, USA) at 4°C overnight. Membranes were then shaken with secondary antibodies for 1 hour at room temperature. Signals were obtained using ECL reagent and detected with UVITEC imaging system equipment. The relative protein expression of p-NF-*κ*B and NF-*κ*B, p-p-38 and p-38, and p-JNK and JNK (relative to GAPDH) from western blot data was determined using ImageJ.

### 2.6. Cell Viability Assay

The MTT (3-(4, 5-dimethylthiazol-2-yl)-2, 5-diphenyltetrazolium bromide) assay was performed to determine cell viability [3]. HaCaT cells were seeded at a density of 10^4^ per 100 *μ*l culture media in 96-well plates. *B. davidii* aqueous extract was added at various concentrations from a dilution series from 100 *μ*g/ml, 50 *μ*g/ml, 25 *μ*g/ml, 12.5 *μ*g/ml, 6.25 *μ*g/ml, 3.125 *μ*g/ml, and 1.5625 *μ*g/ml. After incubation for 24 hours, the cells were incubated with a 0.1% MTT solution in the cell culture medium for 4 hours at 37°C in a humidified incubator under 5% CO_2_. The MTT solution was discarded, and DMSO was added to solubilize the MTT-formazan crystals produced in the live cells. After incubating in the dark for 2 hours, absorbance was measured at 540 nm [16].

### 2.7. Statistical Analysis

All data are presented as mean ± standard deviation (SD) of at least three independent experiments and were analyzed using one-way ANOVA. Student's *t* test was used when only two groups were compared. Differences were considered statistically significant at *p* < 0.05, *p* < 0.01.

## 3. Results

### 3.1. Screening for Potential Anti-Inflammatory Activity of Aqueous Extract of Plants

The effect of plant aqueous extract on the expression of genes is given in Table 2.

The inflammatory process increases the expression of COX-2 and NO synthase, which produce inflammatory mediators [12, 17]. In addition, proinflammatory cytokines such as TNF-*α*, IL-1*β*, IL-6, and IL-8 trigger and rapidly amplify the inflammatory response to limit the spreading of the infection [2, 18]. In this study, 16 plants were extracted from Jeju Island (Korea) that were tested for their anti-inflammatory activity against *C. acnes*-induced inflammation. The percentage of gene expression described in Table 2 was calculated based on the ratio of gene expression of *C. acnes*-infected HaCaT cells treated with aqueous plant extract over one without the plant aqueous extract as an increasing 100% compared with *C. acnes*-free HaCaT cells. Reduced values represent the inhibition of the expression of COX-2, NO, and proinflammatory cytokines such as TNF-*α*, IL-1*β*, IL-6, and IL-8. Table 2 shows *A. japonica* A. Gray, *B. davidii*, *I. verum*, and *P. fibrosa* aqueous extract decreased the inflammation process caused by *C. acnes*. Among them, *B. davidii* aqueous extract showed the strongest inhibition toward the inflammatory process caused by *C. acnes* via reduced iNOS, COX-2, TNF-*α*, IL-1*β*, IL-6, and IL-8 expression (Table 2).

### 3.2. Suppression of C. acnes-Induced TLR-2 Expression in HaCaT Cells by B. davidii Aqueous Extract

**Figure 1:**
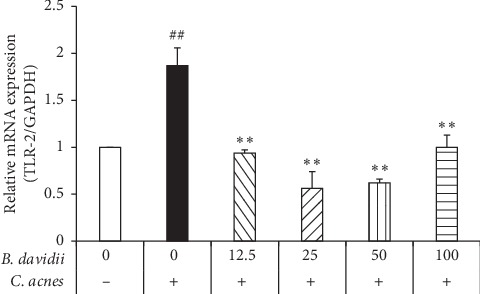
*B. davidii* aqueous extracts inhibited the expression of TLR-2 in *C. acnes*-treated HaCaT cells. The expression level of TLR-2 mRNA was determined using a RT-PCR. HaCaT cells were seeded in six-well plates for 24 hours and then were pretreated with various concentrations (12.5, 25, 50, and 100 µg/ml) of *B. davidii* aqueous extracts for 1 hour and incubated with heat-killed *C. acnes* (100 MOI) for 24 hours. The values are expressed as the mean ± SD of three independent experiments, ^##^*p* < 0.01 vs *C. acnes*-free and without *B. davidii* aqueous extract, ^*∗*^*p* < 0.05, ^*∗*^*p* < 0.01 vs *C. acnes* and without plants aqueous extract.

*C. acnes* contribute to the inflammation in acne through activation of toll-like receptors (TLRs), in particular, TLR-2 [2, 19]. In the initial screening, *B. davidii* aqueous extracts displayed the strong decline of the proinflammatory cytokine expressions in *C. acnes*-treated HaCaT cells (Table 2). The effects of *B. davidii* aqueous extract on expression of the pattern recognition receptor TLR-2 showed *C. acnes* induced the expression of TLR-2 while treated with 50 *μ*g/ml of *B. davidii* aqueous extracts and reduced the TLR-2 mRNA expression by 30% compared with *C. acnes*-treated HaCaT cells without *B. davidii* aqueous extract (Figure 1).

### 3.3. B. davidii Aqueous Extract Reduced NF-kB Phosphorylation in C. acnes-Treated HaCaT Cells

**Figure 2:**
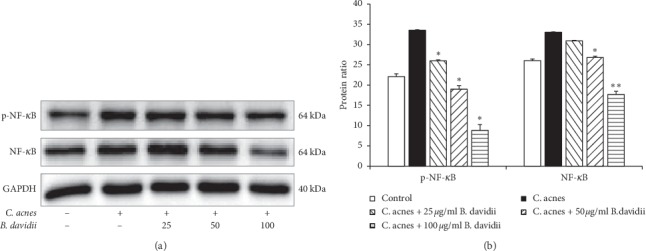
Reduction of the protein ratio of NF-*κ*B in *C. acnes*-treated HaCaT cells by *B. davidii* aqueous extracts. HaCaT cells were pretreated with the indicated concentration of *B. davidii* aqueous extracts for 1 hour before treatment with heat-killed *C. acnes* (100 MOI) for 24 hours. Western blot analysis was carried out to evaluate the level of phosphorylated NF-*κ*B or NF-*κ*B. (a) *B. davidii* aqueous extracts reduced the phosphorylation of NF-*κ*B and *C. acnes*-induced inflammation. (b) Relative protein expression of p-NF-*κ*B and NF-*κ*B (relative to GAPDH) was determined using densitometry. Control was *C. acnes*-free without *B. davidii* aqueous extract. All data were expressed as mean ± SD. ^*∗*^*p* < 0.05 compared with *C. acnes* treated cells only.

TLR-2 signaling leads to the activation of the NF-*κ*B pathway, and then NF-*κ*B is released and translocates to the nucleus where it regulates expression of an array of genes, which in turn stimulates the release of proinflammatory cytokines such as IL-1*β*, IL-6, IL-8, and TNF-*α* [2–4]. The NF-*κ*B pathway was activated by *C. acnes,* indicated by increased expression of toll-like receptor 2 (TLR-2) (Figure 1) compared with *C. acnes*-free. *B. davidii* aqueous extract dose-dependently reduced the amount of p-NF-*κ*B and NF-*κ*B protein which could be due to the decrease of the gene expression, as determined using western blotting (Figure 2). These results suggest that the anti-inflammatory activity of *B. davidii* aqueous extract in HaCaT cells with *C. acnes*-induced inflammation might be due to suppression of the NF-*κ*B signaling pathway.

### 3.4. Inhibition of C. acnes-Induced p38 and JNK Expression and Phosphorylation by B. advidii Aqueous Extract

**Figure 3:**
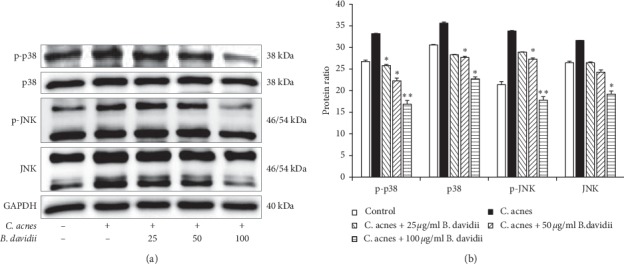
Inhibition of *B. davidii* aqueous extract on MAPKs expression in *C. acnes*-treated HaCaT cells. Western blot analysis was used to check the amount of protein of p-p38, p38, p-JNK, and JNK. HaCaT cells were pretreated with the indicated concentration of *B. davidii* aqueous extracts for 1 hour before treatment with heat-killed *C. acnes* (100 MOI) for 24 hours. (a) Total protein expression and phosphorylation of p38 and JNK were reduced by treatment with *B. davidii* aqueous extracts. (b) Relative protein expression of p-p38, p38, p-JNK, and JNK (relative to GAPDH) was determined using ImageJ. All data were expressed as mean ± SD. ^*∗*^*p* < 0.05, ^*∗∗*^*p* < 0.01 compared with *C. acnes*-treated cells only.

The mitogen-activated protein kinase (MAPK) signaling pathways play an essential role in regulating the production of multiple inflammatory mediators [2, 4, 20]. The effects of *B. davidii* aqueous extract on *C. acnes*-induced MAPK pathway activity were examined using western blotting for p-JNK and p-p38 (Figure 3). *C. acnes*-induced MAPK activity in HaCaT cells and treatment with *B. davidii* aqueous extract extracts dose-dependently suppressed this MAPK activity. These results indicate that *B. davidii* aqueous extract exerts its anti-inflammatory actions via inhibition of the MAPK signaling pathway.

### 3.5. B. davidii Aqueous Extract Inhibited the C. acnes-Induced Proinflammatory Cytokine Expression-Induced HaCaT Cells

**Figure 4:**
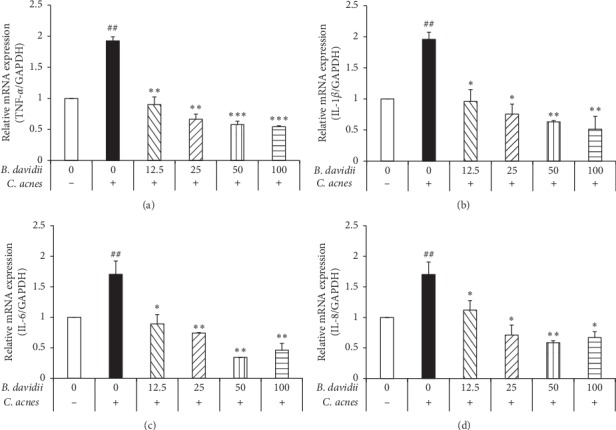
*B. davidii* aqueous extract reduced the level of the proinflammatory cytokines expression in *C. acnes*-treated HaCaT cells. HaCaT cells were pretreated with *B. davidii* aqueous extract (12.5, 25, 50, and 100 *μ*g/ml) 1 hour before incubation with *C. acnes* (100 MOI) for 24 hours. The expression of TNF-*α*, IL-1*β*, IL-6, and IL-8 mRNA was then measured using RT-PCR. Experiments were performed in triplicate using independent samples, and the data are presented as the mean ± SD, ^##^*p* < 0.01 vs *C. acnes*-free and without *B. davidii* aqueous extract, ^*∗*^*p* < 0.05, ^*∗∗*^*p* < 0.01, ^*∗∗∗*^*p* < 0.001 vs *C. acnes* and without plant aqueous extract.

The primary proinflammatory cytokines are IL-1*β*, IL-6, IL-8, and TNF-*α* that play important roles in inflammation [4, 18]. To assess the effects of *B. davidii* aqueous extract on *C. acnes*-induced proinflammatory cytokine expression, HaCaT cells were pretreated with indicated concentrations (12.5, 25, 50, and 100 *μ*g/ml) of *B. davidii* aqueous extract for 1 hour prior to addition of heat-killed *C. acnes* (100 MOI) for 24 hours. *C. acnes*-treated HaCaT increased the expression of IL-1*β*, IL-6, IL-8, and TNF-*α*. However, the expression of IL-1*β*, IL-6, IL-8, and TNF-*α* was dose-dependently declined in *C. acnes*-treated cells after treatment with *B. davidii* aqueous extract (Figure 4). IL-1*β*, IL-6, IL-8, and TNF-*α* expression was decreased by approximately 32%, 21%, 35%, and 29% at 50 *μ*g/ml, respectively, compared with untreated cells. Hence, 50 *μ*g/ml of *B. davidii* aqueous extract is the highest concentration of the extract shows the sharpest effect. These results indicate that *B. davidii* aqueous extract could have anti-inflammatory effects through inhibiting proinflammatory cytokine expression.

### 3.6. Cytotoxicity of B. davidii Aqueous Extract in HaCaT C

**Figure 5:**
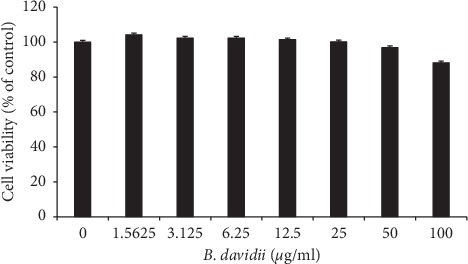
Effects of *B. davidii* aqueous extracts on HaCaT cell viability. HaCaT cells (10^4^ per well) were incubated with *B. davidii* aqueous extracts in 96-well plates for 24 hours, and cell viability was evaluated using the MTT assay. Data represent three independent experiments.

To determine the cytotoxic effects of *B. davidii* aqueous extracts on HaCaT cells, the MTT assay was used. The cells were treated with *B. davidii* aqueous extracts for 24 hours at a concentration ranging from 0.39 *μ*g/ml to 100 *μ*g/ml, and the *B. davidii* aqueous extracts had no significant cytotoxic effects on HaCaT cells (Figure 5).

## 4. Discussion

Inflammation has been suggested as a key factor involved in the development and aggravation of acne vulgaris, although the exact pathogenesis of acne vulgaris has not been elucidated. It has been verified that *C. acnes* play a major role in the development of inflammatory acne lesions [1, 4]. *C. acnes* promote the activation of TLR-2 in keratinocytes, aggravating the inflammation reactions [2, 4]. In fact, the *C. acnes* infection begins to activate monocyte TLR-2, a major signaling pathway induced by activation of NF-*κ*B or MAPKs signaling pathway, which in turn gives rise to expression of cytokines, chemokines, adhesion molecules, and granulopoiesis factors [4, 18, 20]. On the basis of this information, *B. davidii* aqueous extract effectively prevents the inflammation caused by *C. acnes* through inhibition of the TLR-2 to NF-*κ*B signaling cascade in keratinocytes (Figures 1 and 2).

**Figure 6:**
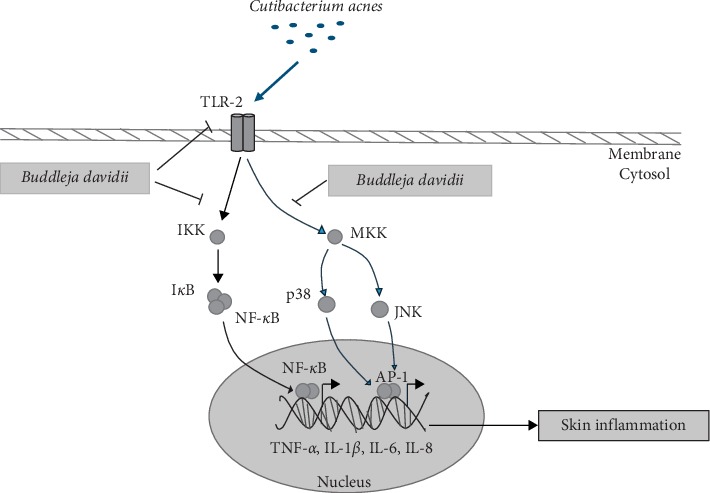
A proposed mechanism of *B. davidii* against *C. acnes*-induced skin inflammation. *B. davidii* inhibits the inflammation by suppressing the NF-*κ*B and MAPK signaling pathways, which are activated by TLR-2, leading to the reduced expression of the proinflammatory cytokines TNF-*α*, IL-1*β*, IL-6, and IL-8.

Recently, it has been studied that the MAPK signaling pathway plays a critical role in regulation of the inflammatory response and coordination of the induction of many genes encoding inflammatory mediators [13, 19, 20]. *B. davidii* aqueous extract suppressed mitogen-activated protein kinase (MAPK) by inhibiting p-p38 and p-JNK in HaCaT cells treated with *C. acnes* (Figure 3). Activation of the TLR-2-mediated downstream MAPK signaling pathway is responsible for production of inflammatory cytokines. MAPK activation has been found in clinical acne lesions, indicating that inhibition of MAPK signaling is important for the pathogenesis of acne vulgaris in general [4, 20]. Use of MAPK inhibitors is emerging as an attractive alternative to anti-inflammatory drugs because they decrease synthesis of proinflammatory cytokines [20]. The results of this study suggest that *B. davidii* aqueous extract modulates the expression of proinflammatory cytokines (TNF-*α*, IL-1*β*, IL-6, and IL-8) in the inflammation process through the inhibition of the NF-*κ*B and MAPK kinase pathways (Figures 4 and 6). Hence, *B. davidii* aqueous extract might be a potential MAPK inhibitor to treat the inflammation caused by bacterial infections.

Previously, *R. coreanus*, *S. mukorossi*, and *P. orientalis* aqueous extract had been reported to be anti-inflammatory [21–23]; however, in this study, *R. coreanus*, *S. mukorossi*, and *P. orientalis* aqueous extract did not exhibit their anti-inflammatory activity (Table 2). In addition, *A. japonica* A. Gray, *B. davidii*, *I. verum*, and *P. fibrosa* aqueous extract showed an inhibition potential to the inflammation caused the *C. acnes* (Table 2). Here, *B. davidii* aqueous extract showed a stronger inhibition toward the inflammatory process caused by *C. acnes* than *I. verum* which was reported in the previous studies [15], *A. japonica* A. Gray, and *P. fibrosa* (Table 2).

*B. davidii* is a well-known, traditional, herbal medicine and a promising candidate for the treatment of acne and other inflammatory diseases [6, 7]. A better understanding of the mechanism of *B. davidii*'s anti-inflammatory activity would not only offer insight into inflammation homeostasis but also provide opportunities for the discovery of new anti-inflammatory compounds with fewer side effects in the future.

In conclusion, this study provides the first evidence that *B. davidii* has the anti-inflammation property through inhibiting two pathways that are NF-*κ*B and MAPK kinase, leading to the reduced proinflammatory cytokine expression. These findings suggest that *B. davidii* is a potential effective therapeutic agent for the treatment of acne vulgaris. However, further studies are needed for a full understanding of its mechanism of action as well as identification of the active components of *B. davidii* in anti-inflammatory activities such as luteolin, quercentin, hesperetin, and colchicine [6, 24]. In addition, evaluation of the in vivo effects of *B. davidii* is required for use in clinical applications.

## Figures and Tables

**Table 1:** RT-PCR primer sequences used in this study.

Genes	Forward	Reverse	Reference
iNOS	TTTCCTTACGAGGCGAAGAAGG	GTTAGGAGGTCAAGTAAAGGGC	[11]
COX-2	GCTTCCATTGCCAGAGCAGGCA	GAGCTCTGGATCTGGAACACTG	[12]
TLR-2	TGTCTTGTGACCGCAATGGT	GTTGGACAGGTCAAGGCTTT	[3]
TNF-*α*	TGAGCACTGAAAGCATGATCC	ATCACTCCAAAGTGCAGCAG	[13]
IL-1*β*	TCTTTGAAGAAGAGCCCGTCCTC	GGATCCACACTCTCCAGCTGCA	[14]
IL-6	AGAGTAGTGFAGGAACAAGCC	TACATTTGCCGAAGAGCCCT	[15]
IL-8	GCAGTTTTGCCAAGGAGTGCT	TTTCTGTGTTGGCGCAGTGTG	[3]
GAPDH	GTGAAGGTVGGAGTVAACG	TGAGGTCAATGAAGGGGTC	[16]

**Table 2:** Effect of plant aqueous extract on the expression of genes related to inflammatory mediators and proinflammatory cytokines in *C. acnes*-treated HaCaT cells (unit: %).

Herb	iNOS	COX-2	TNF-*α*	IL-1*β*	IL-6	IL-8
*Akebia quinata*	160	218	81	123	131	87
*Angelica japonica* A. Gray	67	26	58	65	38	87
*Artemisia campestris*	38	53	44	73	126	105
*Buddleja davidii*	41	57	29	32	21	35
*Ceramium kondoi* Yendo	173	148	115	151	117	95
*Chelidonium majus* L. var*. asiaticum* Ohwi	61	54	90	173	112	121
*Elaeagnus glabra*	50	48	114	211	180	183
*Fallopia japonica* (Houtt.) Ronse Decr.	167	238	72	87	109	114
*Hydrangea macrophylla*	120	106	18	22	97	25
*Hydrangea serrate*	178	116	86	111	132	161
*Hydrangea serrata* Seringe	110	90	75	66	83	66
*Illicium verum*	16	73	31	46	61	44
*Platycladus orientalis*	156	114	31	122	15	182
*Pyllacantha fibrosa*	25	77	69	62	36	69
*Rubus coreanus*	60	58	95	113	145	179
*Sapindus mukorossi*	102	99	60	169	127	107

Control 1: HaCaT with *C. acnes*-free and without plants aqueous extract. Control 2: HaCaT with *C. acnes* and without plant aqueous extract (increasing 100% compared with control 1).

## Data Availability

No data were used to support this study.
